# Mouth Hygiene and Mouth Sepsis

**Published:** 1912-10-15

**Authors:** 


### BIBLIOGRAPHY.

MOUTH HYGIENE AND MOUTH SEPSIS. This is the title of a recent contribution to our permanent literature by Dr. John S. Marshall, the Author of the well known works on Operative Dentistry and Oral Surgery. The book is published by The J. B. Lippincott Co. of Philadelphia. The author has attempted to supply a book that will meet

---

3Oliver, Sir Thomas: Bulletin of the Bureau of Labor, July, 1911.

the needs of the general public and at the same time can be used as a text book in our colleges, and as a work of reference for practitioners. This is a most commendable idea, but like all universal tools, its efficiency is seriously handicapped by the effort. In his endeavor to make plain the nomenclature, for instance the author uses the technical term and interjects in parenthesis a translation into the popular vernocular. This makes the book difficult to read consecutively by the dentists and interfers with its narative style, which would help to claim the attention and hold the interest of the layman. This is not however a serious objection, because we shall in due time have more scientific works an the subject, and Dr. Marshall has done us a great service in supplying a book that will tide us over the pioneer period of oral hygiene. The book is timely as it will be most useful in supplying material for the oral hygiene propoganda that is just now absorbing professional men as well as the general public. The book discusses under three general divisions; first "Mouth Hygiene," in more or less popular style of treatment: then the "Pathologic and Therapeutic" conditions under "Oral Sepsis" and thirdly; the public propaganda for "Oral Hygiene" and the results of experiments thus far made in this cause. The work is timely and can most profitably find a place in the library of every dentist and worker in the field of sanitary reforms.

The book is convenient in size and inexpensively printed so that it ought to have a large circulation. The chapter on the care of infants and children's teeth ought to sell the book.

There are numerous typographical errors, and in places hasty preparation is evident, which will undoubtedly receive attention in future editions.

